# ADGRE1 (EMR1, F4/80) Is a Rapidly-Evolving Gene Expressed in Mammalian Monocyte-Macrophages

**DOI:** 10.3389/fimmu.2018.02246

**Published:** 2018-10-01

**Authors:** Lindsey A. Waddell, Lucas Lefevre, Stephen J. Bush, Anna Raper, Rachel Young, Zofia M. Lisowski, Mary E. B. McCulloch, Charity Muriuki, Kristin A. Sauter, Emily L. Clark, Katharine M. Irvine, Clare Pridans, Jayne C. Hope, David A. Hume

**Affiliations:** ^1^The Roslin Institute, University of Edinburgh, Edinburgh, United Kingdom; ^2^Nuffield Department of Clinical Medicine, John Radcliffe Hospital, University of Oxford, Oxford, United Kingdom; ^3^Royal (Dick) School of Veterinary Studies, University of Edinburgh, Edinburgh, United Kingdom; ^4^Mater Research-University of Queensland, Woolloongabba, QLD, Australia; ^5^Centre for Inflammation Research at the University of Edinburgh, Edinburgh, United Kingdom

**Keywords:** macrophage, monocyte, bone marrow, F4/80, porcine, ADGRE1/EMR1, adhesion G protein-coupled receptor E1

## Abstract

The F4/80 antigen, encoded by the *Adgre1* locus, has been widely-used as a monocyte-macrophage marker in mice, but its value as a macrophage marker in other species is unclear, and has even been questioned. ADGRE1 is a seven transmembrane G protein-coupled receptor with an extracellular domain containing repeated Epidermal Growth Factor (EGF)-like calcium binding domains. Using a new monoclonal antibody, we demonstrated that ADGRE1 is a myeloid differentiation marker in pigs, absent from progenitors in bone marrow, highly-expressed in mature granulocytes, monocytes, and tissue macrophages and induced by macrophage colony-stimulating factor (CSF1) treatment *in vivo*. Based upon these observations, we utilized RNA-Seq to assess the expression of *ADGRE1* mRNA in bone marrow or monocyte-derived macrophages (MDM) and alveolar macrophages from 8 mammalian species including pig, human, rat, sheep, goat, cow, water buffalo, and horse. *ADGRE1* mRNA was expressed by macrophages in each species, with inter-species variation both in expression level and response to lipopolysaccharide (LPS) stimulation. Analysis of the RNA-Seq data also revealed additional exons in several species compared to current Ensembl annotations. The ruminant species and horses appear to encode a complete duplication of the 7 EGF-like domains. In every species, Sashimi plots revealed evidence of exon skipping of the EGF-like domains, which are highly-variable between species and polymorphic in humans. Consistent with these expression patterns, key elements of the promoter and a putative enhancer are also conserved across all species. The rapid evolution of this molecule and related ADGRE family members suggests immune selection and a role in pathogen recognition.

## Introduction

The F4/80 antigen, first identified by Austyn and Gordon (1), was rapidly adopted as a marker for cells of the mononuclear phagocyte lineage in mice (2). F4/80 expression varies amongst mouse mononuclear populations, being very low or absent from osteoclasts, macrophages of T cell areas and marginal zone, lung alveolar macrophages, and the majority of classical dendritic cells [reviewed in (3)]. High expression of F4/80 has been proposed as a marker for populations of mouse tissue macrophages that derive from embryonic progenitors and renew independently of blood monocytes (4). The isolation of F4/80 cDNA (5) revealed that it encodes a large extracellular domain containing multiple Epidermal Growth Factor (EGF)-like calcium-binding domains, linked to a seven-transmembrane domain characteristic of G protein coupled receptors. The mouse gene encoding the F4/80 antigen was given the name *Emr1* (EGF module-containing mucin-like receptor) based upon homology to a previously-identified human cDNA (6). Subsequent studies identified related receptors (*EMR2, EMR3, EMR4, CD97*) collectively called EGF-TM7 proteins (5). These receptors are a subfamily of adhesion G protein coupled receptors (ADGRE) (7) and *Emr1* has been renamed *Adgre1*. Despite the high level of expression and lineage restriction in mouse mononuclear phagocytes, the knockout of *Adgre1* in the mouse germ line produced few phenotypic impacts, apart from apparent dysregulation of autoimmune responses and generation of regulatory T cells (8). One possible explanation for the lack of phenotype in mutant mice is the existence of a closely-related gene, *Adgre4* (EMR4), which is also expressed in mouse monocyte-macrophage lineage cells (9, 10). *Adgre1* mRNA is also highly-expressed by rat macrophages (11, 12).

Rodent macrophages differ significantly from humans in both constitutive and inducible gene expression profiles (13–15). In humans, ADGRE1 has been proposed as an eosinophil-specific marker. Monoclonal antibodies produced against the human protein bound specifically to eosinophils, and *ADGRE1* mRNA was apparently enriched in these cells compared to mononuclear phagocytes (16, 17). F4/80 antibody also binds to mouse eosinophils (18, 19). *Adgre1* mRNA in mice is also expressed by neutrophils, although the protein is not present on the cell surface (20). The apparent lack of *ADGRE1* in human monocyte-macrophages is not likely to be compensated by *ADGRE4* which was annotated as a pseudogene, due to a one base deletion which alters the reading frame before the transmembrane domains. The *ADGRE4* transcript, if expressed, could potentially encode a secreted extracellular domain, with the open reading frame encoding the seven-transmembrane domain retained intact and containing a start codon, and so is potentially translated separately (21). However, by contrast to rodents, the human genome contains two additional members of the EGF-TM7 family, *ADGRE2* and *ADGRE3*, which are both highly-expressed in myeloid cells (22). The receptors encoded by these genes might fulfill functions mediated by ADGRE1 in rodents.

We have previously characterized peripheral blood monocytes and macrophages in the domestic pig and shown that pigs resemble humans in their patterns of constitutive and inducible gene expression (23–25). Pigs are increasingly recognized as a superior predictive model for the innate immune system and the pathology of human disease (26). We showed previously that *ADGRE1* mRNA is highly-expressed in several macrophage-rich pig tissues, and strongly-induced in the liver, associated with macrophage infiltration, following treatment with macrophage colony-stimulating factor (CSF1) (27). CSF1 treatment of mice was shown to increase the level of F4/80 on individual macrophages (28) and *Adgre1* mRNA was also strongly-induced in the liver of rats treated with CSF1 (12). Here we describe the production and characterization of an anti-pig ADGRE1 monoclonal antibody. The antibody binds strongly to monocytes and granulocytes in bone marrow and blood, and to tissue macrophages.

Our laboratory has also generated RNA-Seq expression profiles of CSF1-stimulated macrophages from rats and from multiple large animal species, including pig, sheep, goat, cow, water buffalo, and horse (29, 30). There are numerous human macrophage RNA-Seq datasets available in the public domain. Based upon the macrophage-enriched expression in the pig, we (re)examined the expression and complex alternative splicing of *ADGRE1* in each of these species. We conclude that *ADGRE1* is highly-expressed and regulated in macrophages in large animals, but that both the protein-coding sequence and the transcriptional regulation is divergent amongst species. We discuss the rapid evolution of these genes in mammals and speculate on their possible function.

## Materials and methods

### Ethics statement

Approval for this research was obtained from The Roslin Institute and University of Edinburgh Protocols and Ethics Committees. All experiments were carried out under the authority of a UK Home Office Project Licence under the regulations of the Animals (Scientific Procedures) Act 1986.

### Primary cell isolation and culture

Isolation of primary pig bone marrow, peripheral blood mononuclear cells (PBMCs) and alveolar macrophages was carried out as described previously (24, 25) and all cells were cultured in full RPMI-1640 medium containing 10% HI-FCS (Sigma), 1 mM Glutamax, 100 U/ml penicillin and 100 μg/ml streptomycin (Invitrogen). Primary pig bone marrow cell cultures were supplemented with 10^4^ Units/ml rh-CSF1 to produce bone marrow-derived macrophages (BMDM) as described elsewhere (24).

### *In-vivo* treatment with pCSF1-Fc recombinant protein

Large white pigs were treated with pCSF1-Fc recombinant protein as previously described (27). In summary 8.5 week old animals were injected subcutaneously on three consecutive days with pCSF1-Fc (0.75 mg/kg; *n* = 6) or PBS control (*n* = 5). Blood was taken and all animals weighed 4 days prior to the first injections. Animals were euthanized by captive bolt following sedation with ketamine and azaperone, following which cells of interest were isolated and frozen for future analysis. Cells from a subset of these animals were utilized herein.

### Construction of *Sus scrofa* ADGRE1 chimeric expression vector

The full-length cDNA sequence of *Sus scrofa ADGRE1* (Ensembl *S. scrofa* 10.2) was amplified using the primers fwd 5′-GGTCCTCACTCAATCTGCAAG-3′ and rev 5′-GGAACAGCATTTTGGAAAGC-3′, then cloned into pGEM®-T Easy vector (Promega). The cDNA encoding the four N-terminal extracellular EGF-like domains (1,082 bp) was extracted from the pGEM®-T Easy vector by PCR then cloned in frame into the EcoRI-EcoRV site of the pFUSE–hIgG1-Fc2 vector (InvivoGen) and sequenced by Edinburgh Genomics. We note that subsequent to our study, there are now sequences for pig *ADGRE1* (GACC01000182.1/JAA53625.1) and *ADGRE4* (GACC01000180.1/JAA53627.1) mRNA and proteins in the TSA archives.

### Recombinant protein production

HEK293T cells grown in DMEM media supplemented with 10% Ig-depleted HI-FCS (Sigma), 1 mM Glutamax, 100 U/ml penicillin and 100 μg/ml streptomycin (Invitrogen) were transiently transfected with pADGRE1-pFUSE-hIgG1-Fc2 DNA at a ratio of 1:1 with Lipofectamine 2000 (Life Technologies) following manufacturer's instructions. Four days post-transfection, the supernatant was harvested, centrifuged to remove debris and filter sterilized. Soluble protein was then purified using a HiTrap Protein G column (GE Healthcare) and desalted using Slide-A-Lyzer™ G2 Dialysis Cassettes 1–3 mL 10 K MWCO (Life Technologies). The recombinant protein was used as an immunogen for monoclonal antibody (mAb) production.

### Monoclonal antibody production

BALB/c mice were purchased from Charles River Laboratories (UK). Hybridoma production was performed as previously described (31). In brief, three BALB/c female mice received a total of three subcutaneous immunisations with 50 μg of pADGRE1-hIgG1-Fc2 recombinant protein and TiterMax Gold adjuvant (Sigma) at least 14 days apart. Immune response was determined by screening sera collected from all mice prior to immunization and after the second and subsequent injections by indirect ELISA. A final intraperitoneal immunization of 50 μg protein in PBS without adjuvant was given and 4 days later splenocytes were harvested.

Splenocytes were fused with Sp2/0-Ag14 mouse myeloma cells (32) at a ratio of 5:1 using polyethylene glycol 1500 (Sigma), following standard procedures. Following fusion, fused hybridoma cells were grown in RPMI-1640 media supplemented with 10% IgG-depleted HI-FCS, 1 mM Glutamax, HAT Media Supplement Hybri-Max™ (Sigma), Hybridoma Fusion and Cloning Supplement (HFCS, Roche), recombinant mouse IL-6 (Sigma), 100 U/ml penicillin, and 100 μg/ml streptomycin. After seven to 10 days cells were switched to RPMI-1640 containing 10% IgG-depleted HI-FCS, 1 mM/l Glutamax, HT Media Supplement Hybri-Max, mouse IL-(6.25 ng/μl)6, 100 U/ml penicillin and 100 μg/ml streptomycin. An indirect ELISA was performed to identify hybridomas producing antibodies against porcine ADGRE1. Positive wells were expanded and subcloned to single cell colonies by limiting dilution. Supernatant was collected and purified by Protein G HiTrap column, with desalting carried out using Slide-A-Lyzer™ G2 Dialysis Cassettes 1–3 mL 10 K MWCO.

The mAbs were isotyped using IsoStrip Mouse Monoclonal Antibody Isotyping Kit (Roche) according to manufacturer's instructions.

### Detection of pig ADGRE1 protein by indirect ELISA

Indirect ELISA was performed as previously described (33). In brief, microplates were coated with 50 μg of 1 μg/ml pADGRE1-pFUSE-hIgG1 or Human IgG1-Fc recombinant protein (R&D Systems) diluted in coating buffer and incubated at 4°C overnight. The following day plates were blocked in PBS, 1% BSA, 1% horse serum for 1 h, followed by three washes with PBS-0.05% Tween20. Fifty microliters of pre- or post-immune serum diluted 1:200 in PBS, neat supernatant from hybridoma cloning plates or 1 μg/ml purified mAb in PBS was added to individual wells and incubated for 1 h at room temperature. Following a further three washes in PBS-0.05% Tween20 50 μl of horse anti-mouse IgG-HRP (Cell Signaling) diluted 1:5,000 in PBS was added and incubated for 1 h at room temperature. After three further washes peroxidase activity was visualized using TMB Substrate (BD Biosciences). The reaction was stopped with 2 N hydrochloric acid. A Multiskan Ascent spectrophotometer was used to read absorbance at 450 nm.

### Flow cytometry

One million cells per sample were first blocked using PBS, 2% normal horse serum for 15 min on ice, then incubated with either neat supernatant for initial screening or a 1:100 dilution of Alexa-Fluor-647 conjugated purified mAb for 30 min on ice. Conjugation was performed using a commercially available kit (Molecular Probes) according to manufacturer's instructions. Signal was detected on unconjugated supernatant by incubation with anti-mouse IgG-allophycocyanin (1:400; BioLegend) for 30 min on ice. Following 3 washes in PBS, cells were re-suspended in 0.1% Sytox Blue (Invitrogen) in PBS for analysis using a Fortessa LSR flow cytometer (BD Biosciences). Double staining included mouse anti-pig CD14-FITC (1:50; BioRad), mouse anti-pig CD16-PE (1:200, BioRad), mouse anti-pig CD163-PE (1:100, BioRad) and mouse anti-pig SIRP-alpha-PE (1:400, Southern Biotech). Relevant isotype controls were included in all experiments. Data collection was performed using Diva software (BD Biosciences) and analysis using FlowJo v10 software. Welch's *t*-tests were performed on data and results are presented as treatment group means ± SE. All statistical analyses were performed using GraphPad Prism 6.0 (GraphPad Software). A *p* < 0.05 was considered statistically significant.

### Immunohistochemistry for ADGRE1 and CD163

Tissues were placed in phosphate buffered saline solution pH7 and mounted in OCT. Frozen sections (10 μm) were cut and mounted on Superfrost slides, then dried for 24 h at 4°C prior to use. The slides were fixed for 10 min in ice cold methanol then washed 2 × 5 min in PBS. To block endogenous peroxidase activity the slides were incubated in 0.3% hydrogen peroxide (H_2_O_2_) in PBS then washed 2 × 5 min in PBS. The slides were incubated for 1 h in a humidified chamber at room temperature in Tris-buffered saline with 20% normal goat serum and 5% BSA, then washed 2 × 5 min in PBS. Immunostaining was performed using primary mAbs against mouse anti-pig ADGRE1 (ROS-4E12-3E6, 2.4 ng/μL) and mouse anti-human CD163 (2 ng/μL, BioRad); control slides had no primary antibody added. Slides were incubated overnight at 4°C then washed 2 × 5 min in PBS. The primary antibodies were detected using anti-mouse IgG polymer (Vector MP-7402) incubated at room temperature for 1 h then washed 2 × 5 min in PBS. The signal was detected using DAB peroxidase substrate (Vector Laboratories SK-4100) then washed 2 × 5 min in water. Sections were counterstained with haematoxylin and eosin using a Leica Autostainer XL. Slides were then mounted, and microscopic analysis performed using the NanoZoomer-XR (Hamamatsu).

### Sources of RNA-seq data

RNA-Seq data was previously generated by our lab for BMDM, or monocyte-derived macrophages (MDM) of rat, cattle, water buffalo, sheep, goat, pig and horse. All RNA-Seq libraries were paired-end, the read length for rat was 90 bp, for goat was 75 bp and for the remainder of the species was 124–126 bp. RNA-Seq data was also available for a subset of these species (sheep, water buffalo and goat) from alveolar macrophages. The sheep data was published as part of a comprehensive transcriptional atlas (34). A subset of data from the remaining species was previously analyzed in a comparative study of the regulation of genes involved in arginine metabolism and nitric oxide production (35), which generated gene-level expression estimates, as transcripts per million (TPM), using Kallisto v0.43.0 (36). The primary RNA-Seq data is available in the European Nucleotide Archive (ENA) using NCBI BioProject IDs PRJEB21180 (water buffalo), PRJEB19199 (sheep), PRJEB22535 (cattle, unpublished), PRJEB23119 (pig), PRJEB24920 (horse), PRJEB22553 (rat, unpublished), and PRJEB23196 (goat). Human alveolar macrophage, microglia, non-classical monocyte, and classical monocyte datasets were obtained from, respectively, Pinilla-Vera et al. (37); 10 RNA-Seq libraries within NCBI BioProject PRJNA428090, representing 6 untreated and 4 LPS-treated samples), Galatro et al. (38); 39 RNA-Seq libraries within NCBI BioProject PRJNA387182), Williams et al. (39); 17 RNA-Seq libraries within NCBI BioProject PRJNA339968), and Mirsafian et al. (40); 1 RNA-Seq library within NCBI BioProject PRJNA264020).

## Results

### Production and characterization of a monoclonal antibody against pig ADGRE1

Bone marrow-derived macrophages and alveolar macrophages from the pig were previously profiled as part of a transcriptional atlas project (41). However, at the time of development of the microarray platform used, the *ADGRE1* locus was not annotated. The predicted pig cDNA was truncated at the 5′ end and missed the start codon. The longest *ADGRE1* cDNA for the pig currently in Ensembl (version 91) predicts a protein of 939 amino acids with an extracellular domain containing 7 EGF-like calcium-binding domains, as in the mouse. The predicted amino acid sequence is only around 60% identical to mouse and almost all the variation resides in these EGF-like domains. The longest human cDNA in Ensembl encodes 6 EGF-like domains. Cross-species alignment of mouse and pig cDNA with the human genome reveals that there is an additional candidate exon encoding a seventh EGF-like domain in the human genome (not shown). We have confirmed the expression of this exon in human macrophage RNA-Seq data as discussed below. Including this exon, the longest human ADGRE1 protein is predicted to be 940 amino acids. A recent study (42) utilized RNA-Seq from pig alveolar macrophages to improve the annotation of genes within the pig immunome. Within that project, the alveolar macrophage data were used to assemble two alternative pig *ADGRE1* cDNAs. As part of the ongoing annotation of the pig genome, we have produced RNA-Seq data from pig BMDM grown in CSF1 and stimulated with LPS (29).

Based upon the gene expression data, and emerging evidence that pigs provide a useful model for the study of human macrophage biology (26), we decided to make an antibody against the pig protein, with the expectation that it could (a) enable studies of *ADGRE1* function in a relevant model, (b) confirm the expression of the ADGRE1 protein in macrophages, and (c) potentially cross-react with human ADGRE1. Previous antibodies against human *ADGRE1* were produced in hamsters, against a cell-expressed ADGRE1-CD97 fusion protein (16) and in mice, against a full length ADGRE1 extracellular domain-Fc fusion protein (17). As an immunogen, we constructed cDNA, expressed and purified a pig ADGRE1-Fc fusion protein, immunized mice, and screened hybridomas for specific binding to the fusion protein, but not to the human IgG recombinant tag. From amongst 29 primary hybridomas, eight were cloned, and each produced clones that bound specifically to the immunogen. One mAb, ROS-4E12-3E6, was selected for further detailed characterization. The remaining hybridoma supernatants were screened for cross-reactivity against human and cattle blood monocytes and sheep alveolar macrophages, but none were cross-reactive to any other species. This is likely a reflection of the extensive sequence divergence between the species.

### Expression of ADGRE1 by pig leukocytes

**Figure 1:**
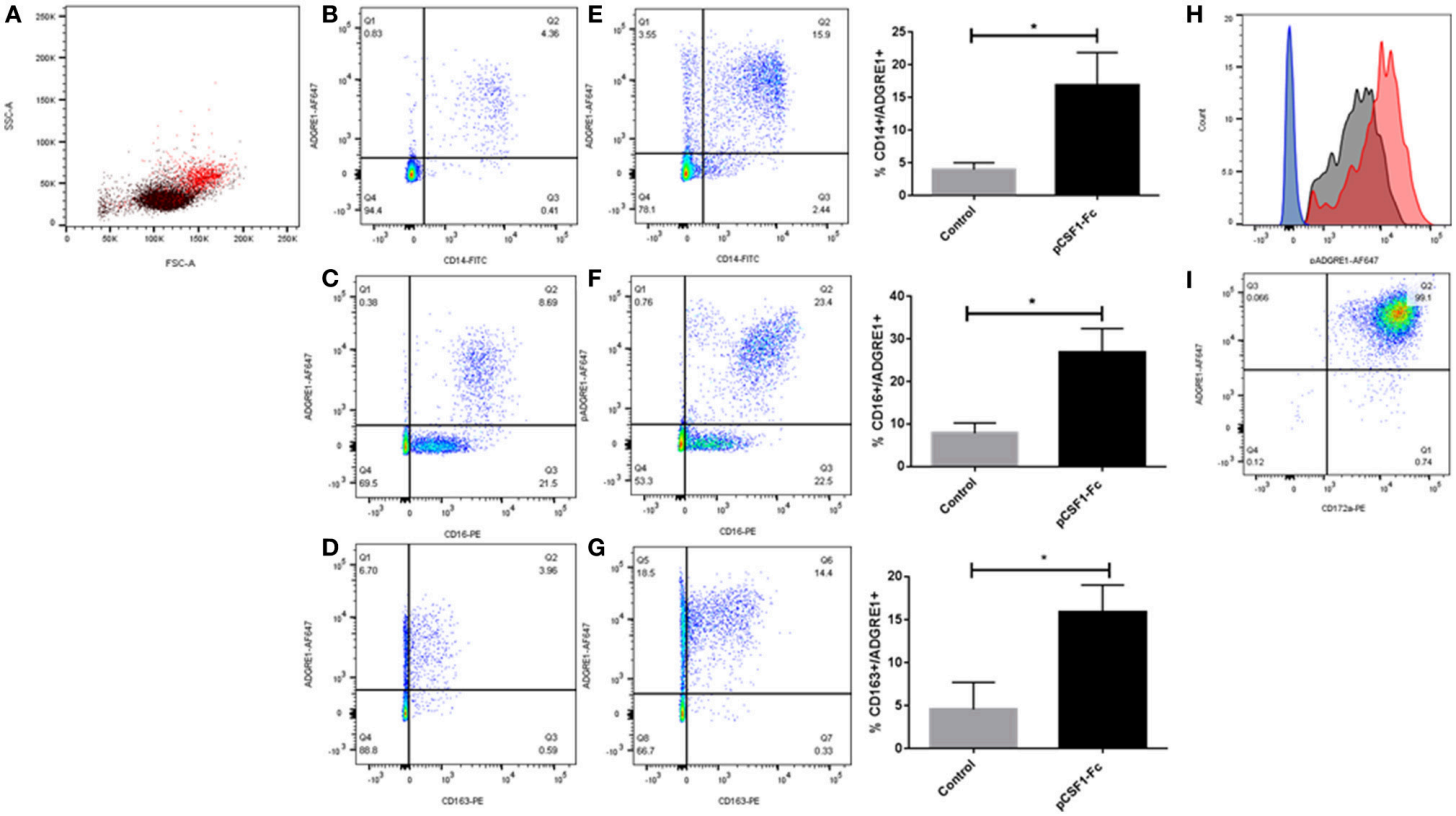
The expression of ADGRE1 on blood leukocytes in the pig. Flow cytometry was carried out on pig PBMCs **(A–H)** and alveolar macrophages **(I)**. Representative flow cytometry images are illustrated (*n* = 3) for live cells (identified by exclusion using Sytox blue). Where shown, quadrant gates were set using isotype matched control antibody staining. Peripheral blood mononuclear cells were examined for expression of ADGRE1 using monoclonal antibody ROS-4E12-3E6 **(A)** and positive cells (red) were identified. Multiple color flow cytometry was used to determine the co-expression of ADGRE1 with CD14 **(B)**, CD16 **(C)**, and CD163 **(D)** on PBMCs. Expression of ADGRE1 with CD14 **(E)**, CD16 **(F)**, and CD163 **(G)** was also determined in PBMC from pigs treated *in-vivo* with pCSF1-Fc treatment. The mean percentage ADGRE1 positive cells ± SE is shown comparing control pigs **(B–D)** with pCSF1-Fc treated pigs **(E–G)**. ^*^P < 0.05 by Welch's *t*-test; *n* = 3 pigs per treatment. Note that these samples are a subset of a larger cohort from our previous study (27) in which CSF1-Fc was shown to increase the blood monocyte count (*P* < 0.0001). **(H)** Histogram of ADGRE1 median fluorescence intensity in PBMC from pCSF1-Fc treated pigs (red), control animals (black), and isotype controls (blue and green for control and treated respectively); *n* = 3. Expression of ADGRE1 was measured on CD172a+ macrophages from bronchoalveolar lavage **(I)**; *n* = 3.

We first examined surface expression of ADGRE1 in PBMC. Figure 1A shows that mAb ROS-4E12-3E6 bound to monocytes, identified in this case by their size and granularity profile. In PBMCs, ADGRE1 was highly-expressed on the entire CD14^++^ monocyte population (Figure 1B). By contrast to humans, where the gene is duplicated, CD16 is not regulated in monocyte subsets in pigs and is uniformly high on the CD14^++^ population (23). Accordingly, ADGRE1 was uniform on all CD16^++^ cells (Figure 1C). The ADGRE1-negative, CD16^lo^ cells are most likely natural killer cells. The haptoglobin receptor, CD163, provides a marker for monocyte differentiation in pigs that varies inversely with CD14, with CD163^hi^ cells resembling intermediate monocytes in humans (23). Here, ADGRE1 expression did not vary with the level of expression of CD163 (Figure 1D).

The differentiation of the non-classical monocyte subset in other species depends upon CSF1 (43) and in mice, F4/80 is also induced further in tissue macrophages following CSF1 treatment (28). We previously developed a porcine CSF1-Fc fusion protein, with an enhanced greater circulating half-life (44). Injection of this protein into mice (44) or pigs (27) led to a substantial increase in blood monocyte and tissue macrophage numbers. Analysis of a subset of samples stored from the previous pig study confirmed that CSF1-Fc treatment significantly increased the percentage of ADGRE1^+^/CD14^+^ blood monocytes (Figure 1E), as well as the proportion of ADGRE1^+^/CD16^++^ (Figure 1F) and ADGRE1^+^/CD163^++^ monocytes (Figure 1G) within the PBMC fraction, when compared to cells from control animals. This result is consistent with the monocytosis described previously and supports the use of ADGRE1 as a pig monocyte marker. The median level of surface ADGRE1 was also increased on the monocytes from CSF1-Fc treated pigs (Figure 1H). In mice, the F4/80 antigen is expressed at very low levels on isolated alveolar macrophages (45). By contrast, and consistent with gene expression profiles reported by Dawson et al. (42), the level of cell surface ADGRE1 was uniformly exceptionally high on pig alveolar macrophages (Figure 1I). The identity of these cells as alveolar macrophages was confirmed by the co-expression of high levels of SIRP-alpha (CD172a), which is highly-expressed by monocytes and macrophages in the pig (23).

**Figure 2:**
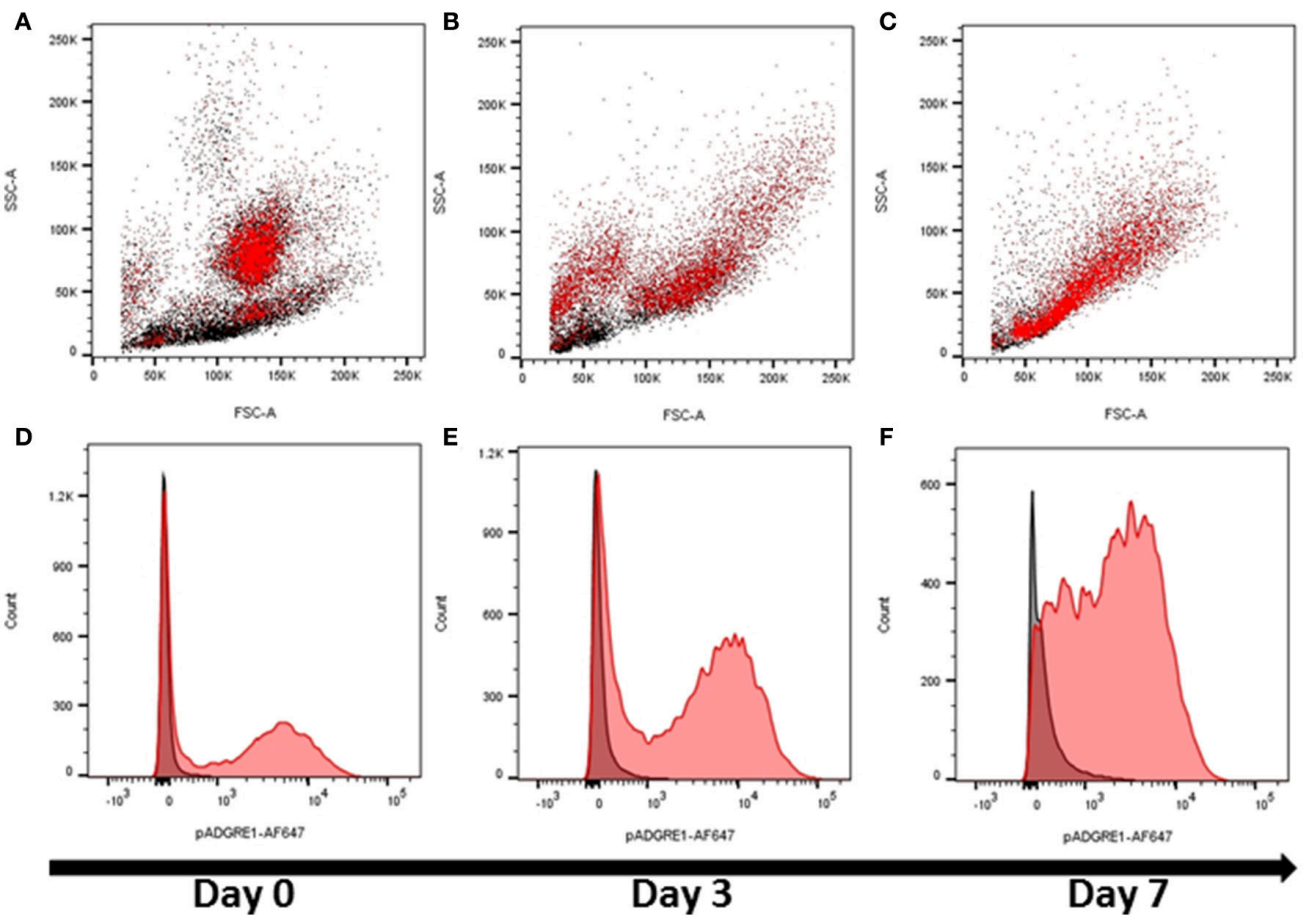
The expression of ADGRE1 in bone marrow progenitors and response to CSF1. Flow cytometry was performed on pig bone marrow cells. Representative flow cytometry images are illustrated (*n* = 3) for live cells (identified by exclusion using Sytox blue). Bone marrow cells were examined for expression of ADGRE1 using monoclonal antibody ROS-4E12-3E6 (red) and the identity of the ADGRE1 positive cells inferred based on size (FSC) and granularity (SSC). ADGRE1 positive cells are shown in red **(A)**. Bone marrow cells were cultivated in rhCSF1 for 3 days **(B)** or 7 days **(C)** and expression of ADGRE1 measured by flow cytometry. The median expression of ADGRE1 was also measured at days 0, 3, and 7 (**D–F**, respectively). Isotype control staining is shown (black line).

These initial observations indicated that ADGRE1 is highly-expressed on the surface of monocytes and macrophages in pigs, but with a distinct pattern compared to rodents. We next considered whether ADGRE1 was widely distributed amongst myeloid cell lineages, and whether it might provide a useful differentiation marker. Figure 2A shows that ADGRE1 was detected on presumptive granulocytes in bone marrow (SSC^hi^). In the bone marrow mononuclear cell fraction, a minor population of larger cells was ADGRE1^hi^, presumably the resident bone marrow macrophages, whereas expression was low and variable on small mononuclear cells. Figure 2D shows the fluorescence histogram, indicating relative uniformity of expression of ADGRE1 amongst the positive cells in unstimulated bone marrow. When bone marrow cells were cultivated in rhCSF1, the ADGRE1^+^/SSC^hi^ granulocyte population largely disappeared within 3 days (Figure 2B), consistent with the short half-life of granulocytes in culture. At the same time, there was an evident expansion of an SSC^lo^, presumptive macrophage population, that had higher levels of ADGRE1 (Figure 2E). After 7 days in rhCSF1 the population of bone marrow cells, largely comprised of adherent macrophages as previously described (24), was entirely SSC^lo^ and ADGRE1^+^ (Figure 2C). At this time, the level of expression of ADGRE1 on individual cells was heterogeneous (Figure 2F).

**Figure 3:**
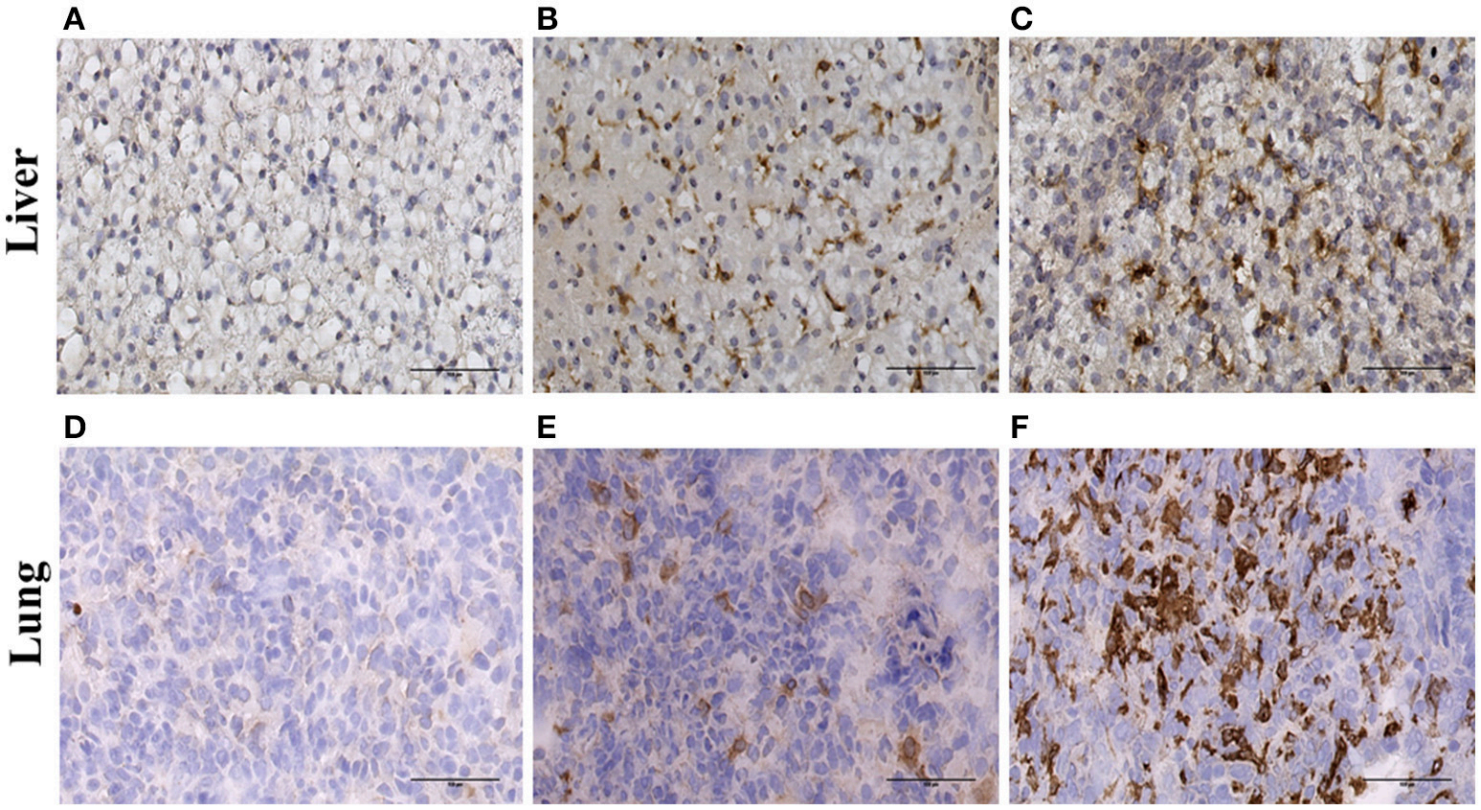
Immunohistochemical localisation of ADGRE1 in pig tissues. Immunohistochemical staining was carried out on frozen pig liver **(A–C)** and lung **(D–F)** tissue. Representative images are shown (*n* = 3). Tissues were incubated without primary antibody (negative control **A,D**), antibody recognizing CD163 (positive control **B,E**) and monoclonal antibody ROS-4E12-3E6 recognizing ADGRE1 **(C,F)**. Scale bar = 100 μm.

To confirm the expression of ADGRE1 on resident tissue macrophages, we stained sections of liver and lung. Compared to the negative control (Figure 3A) and CD163 positive control (Figure 3B) sections there was abundant expression of ADGRE1 on lung macrophages (Figure 3C), including stellate interstitial macrophages and more rounded, presumptive alveolar macrophages. *ADGRE1* mRNA was previously detected in expression profiles of whole pig liver and was massively induced by CSF1-Fc treatment (27). Compared to negative (Figure 3D) and CD163 (Figure 3E) positive sections, high levels of expression of ADGRE1 was also detected on stellate Kupffer cells in pig liver (Figure 3F), showing the same portal-centrilobular gradient observed in mouse liver (46).

### The regulated expression of *ADGRE1* mRNA in human macrophages

Two previous reports used RT-PCR to determine the expression of *ADGRE1* in human myeloid cell populations, and concluded that the mRNA was highly-expressed in eosinophils, and low or undetectable in other leukocyte populations (16, 17). This conclusion was at odds with the detection of the mRNA in mononuclear cells in the original cloning of human *EMR1* cDNA (6). Given the expression of *ADGRE1* in the pig, we concluded that either pigs are fundamentally different from humans, or the previous studies of ADGRE1 expression in humans were incorrect. To address these two possibilities, we examined a range of data available in the public domain. An expression atlas of human cell populations generated based upon public domain microarray data indicates that *ADGRE1* mRNA is expressed at similar levels in neutrophils, monocytes, and alveolar macrophages [(47); see data on www.biogps.org). The FANTOM5 consortium used cap-analysis of gene expression (CAGE, genome-scale 5′ end tag sequencing) to analyze monocyte subsets, monocyte-derived macrophages, activated monocytes and macrophages, and many other myeloid populations (48, 49). The data can be visualized on the ZENBU viewer (http://fantom.gsc.riken.jp/zenbu/). This confirms *ADGRE1* is expressed in human eosinophils, but the level of expression was no higher than in CD16^++^ monocytes. Transcription of *ADGRE1* in all the human myeloid lineages appears to be a differentiation marker, as expected, since mRNA was not detected in CD34^+^ stem cells or committed myeloid progenitors. Data from Maiga et al. (50) also indicate the high expression of *ADGRE1* in monocytes and relative absence from progenitors. The FANTOM5 data also reveal that *ADGRE1* was repressed in MDM grown in CSF1, relative to the higher level of expression in blood monocytes but was strongly induced in MDM relatively late in the time course by LPS. Indeed, the highest expression of ADGRE1 detected in the FANTOM5 dataset was observed in LPS-stimulated cells (30). Comparative analysis of monocytes, MDM and isolated microglia (38) by RNA-Seq confirmed both monocyte expression and relative down-modulation in MDM. The increased expression of *ADGRE1* mRNA in CD16^++^ human monocytes detected by CAGE is confirmed in three published microarray datasets (51, 52). A further marker, SLAN, dissects the CD16^++^ monocyte subpopulation still further, with *ADGRE1* mRNA further elevated in the SLAN-positive population (53). *ADGRE1* mRNA was very highly expressed in human alveolar macrophages (54, 55) and isolated placental macrophages (56) enriched in the macrophage fraction isolated from human adipose tissue (57) and was readily detected in RNA-Seq data derived from human peritoneal macrophages (58). Taken together, all the evidence indicates that *ADGRE1* is robustly expressed in the human macrophage lineage and is not eosinophil-restricted. In humans, the related gene *ADGRE4* is located immediately adjacent to the *ADGRE1* locus. In mice, this gene, also known as FIRE (9, 10), is located around 1.5 mb downstream of *ADGRE1* on chromosome 17. Human *ADGRE4* is annotated as a pseudogene, based upon the analysis of a cDNA that contains a 1 bp deletion relative to the *ADGRE4* gene in other primates, which shifts the reading frame before the transmembrane domain (21, 59). The FANTOM5 data places the transcription start site (TSS) around 7 kb upstream of the published cDNA. Since *ADGRE4* mRNA in other species (mouse, rat, dog, and pig) is around 50-100 bp longer at the 5′ end than human, and since there is one fewer annotated exon compared to mouse, it seems likely that there is an alternative 5′ non-coding exon. *ADGRE4* CAGE tags were even more enriched than *ADGRE1* in CD16^++^ monocytes, compared to CD14^++^ monocytes. In common with many other monocyte differentiation markers, the CD14^+^/CD16^+^ intermediate monocytes expressed *ADGRE4* at intermediate levels (49). Hence, *ADGRE4* is a novel marker for “non-classical” monocytes in humans. The human genome contains two other genes, *ADGRE2* and *ADGRE3*, related to *ADGRE1* and *ADGRE4* and located in the same region of chromosome 19. Consistent with previous reports (60, 61) in the FANTOM5 data, *ADGRE2* shows a very similar pattern of regulation in human monocytes and MDM to *ADGRE1*, whereas *ADGRE3* is more highly-expressed and restricted to granulocytes, neutrophils and eosinophils.

### *ADGRE1, ADGRE2, ADGRE3*, and *ADGRE4* expression in macrophages of other species

As part of the generation of transcriptional atlases for several mammalian species, and a comparative analysis of macrophage-expressed genes (29) we have produced deep RNA-Seq data for rat, sheep, goat, cattle, water buffalo, pig, and horse macrophages produced by the cultivation of bone marrow cells in CSF1 using methods originally developed for the pig (24). We utilized these data to assess the expression of *ADGRE1* and *ADGRE4* and the response to LPS, in each of these species. Table S1 summarizes the expression of *ADGRE1* and the related *ADGRE4* mRNA in BMDM, and the response to LPS, for each of the species examined, including human data from published RNA-Seq profiles. Where available, additional data is shown for isolated alveolar macrophages and for monocyte-derived macrophages. Several findings emerge from these data. Firstly, the highest level of *ADGRE1* mRNA expression detected was in rat BMDM, but *ADGRE1* was robustly expressed in macrophages in all of the species. Secondly, the level and pattern of expression was variable, even between ruminant species. For example, *ADGRE1* was highly-expressed in BMDM from sheep, 10-fold lower in cattle and water buffalo (but LPS-inducible as in human macrophages) and very low in goat. Where we have data available *ADGRE1* was highly expressed in alveolar macrophages in every large animal species. Of the species examined, only the pig had high expression of *ADGRE4* in BMDM. Like the mouse, the rat has only three members of the ADGRE family, *Adgre1, Adgre4*, and *Adgre5* (62) and *Adgre1* mRNA was very highly-expressed in rat BMDM. However, by contrast to mouse, *Adgre4* was barely-detectable in these cells.

*ADGRE2* and *ADGRE3* are not well annotated in livestock species, and clear orthology relationships are unclear. For example, in Ensembl build 11.1, there are now 4 *ADGRE2*-like (ENSSSCG00000036342,ENSSSCG00000021675,ENSSSCG00000013788,ENSSSCG00000013785) and 2 *ADGRE3*-like (ENSSSCG00000013787, ENSSSCG00000026402) genes. In Ensembl, at this time *ADGRE2-* and *ADGRE3*-like genes are annotated as many-to-many orthologs with the other livestock species. We have included expression estimates for these transcripts in pig macrophages in Table S1. Only the two *ADGRE3*-like genes have significant expressions, and then 10-fold lower than *ADGRE1* or *ADGRE4*.

### Evidence of species-specific exon skipping in *ADGRE1*

**Figure 4:**
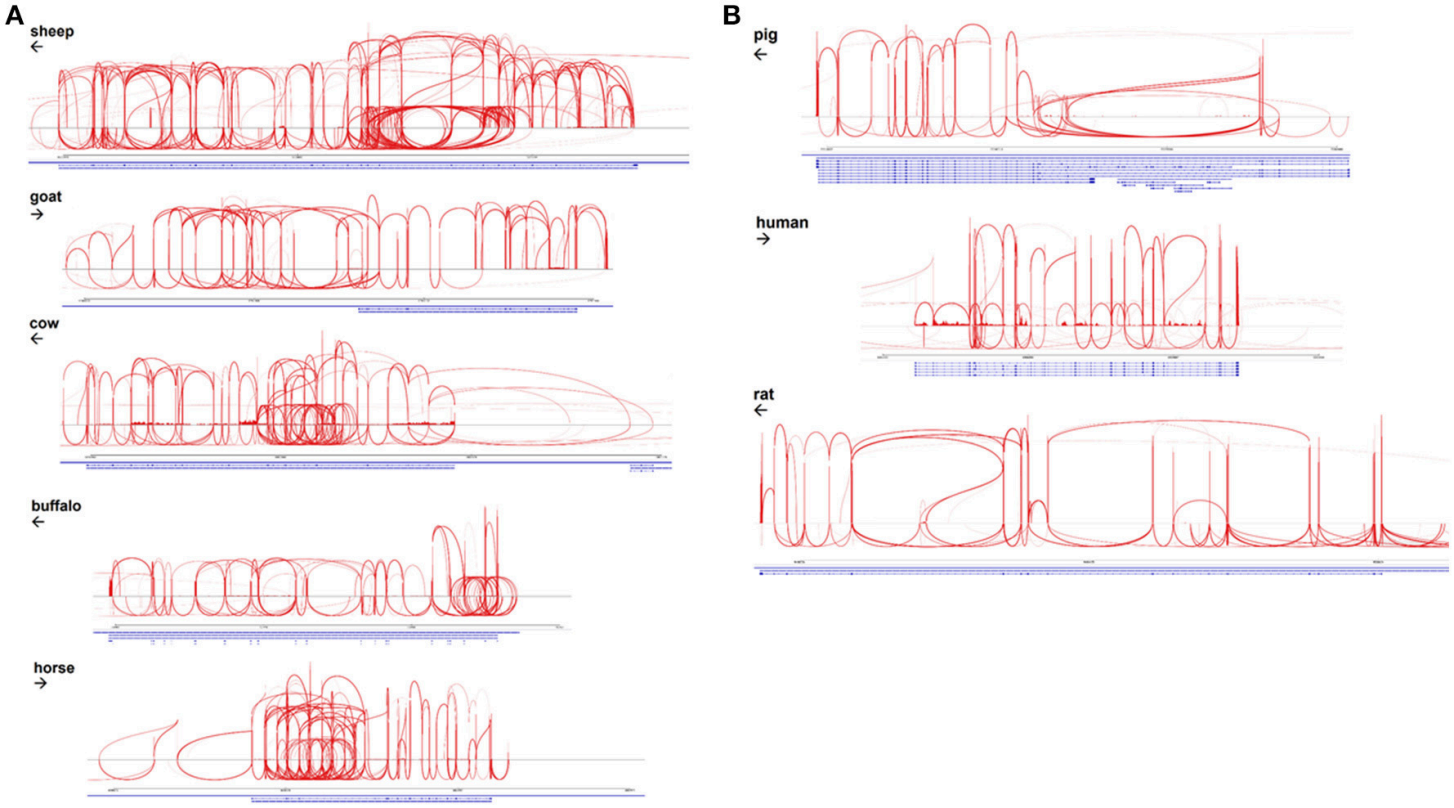
Alternative splicing of *ADGRE1* mRNA in mammalian macrophages. **(A)** Sashimi plots, created using IGV v2.4.10 (35) spanning the *ADGRE1* loci of sheep (annotation Oar v3.1; coordinates 5:15,203,349-15,289,527), goat (ARS1; 7:92,931,693-92,970,027), cattle (UMD3.1; 7:18,767,695-18,834,685), buffalo (UMD_CASPUR_WB_2.0; NW_005785799.1:700,212-743,535), and horse (EquCab2; 7:4,424,464-4,467,628). Arrows indicate the direction of transcription. For each species, RNA-Seq reads mapping to this locus are pooled from all available monocyte and macrophage samples. **(B)** Sashimi plots, created using IGV v2.4.10 (35), spanning the *ADGRE1* loci of pig (annotation Sscrofa11.1; coordinates 2:72,217,039-72,306,013), human (GRCh38.p10; 19:6,887,566-6,940,459), and rat (Rnor_6.0; 9:9,431,860-9,585,865). Arrows indicate the direction of transcription. For each species, RNA-Seq reads mapping to this locus are pooled from all available monocyte and macrophage samples.

The EGF-like calcium binding domains of ADGRE1 are encoded by single exons and can potentially be alternatively spliced. McKnight and Gordon (63) described 5 isoforms of mouse, and 4 isoforms of human ADGRE1, in which variable numbers of EGF-like domains are expressed, from none to 7 in mouse, and 1 to 6 in human. These authors did not report the expression of the seventh EGF-like domain in humans. With the deep RNA-Seq data available for multiple species we examined the extent of alternative splicing in each species by aggregating available macrophage datasets, as shown in Sashimi plots in Figures 4A,B. These data indicate that the pig and human genomes, like the rat, encode a maximum of 7 EGF-like transmembrane domains. The accurate mapping of the RNA-Seq outputs to exons, and especially analysis of splice junctions, is complicated by the presence of an internal duplication in each of these species. The longest predicted cDNA for any species in Ensembl is for sheep, but the RNA-Seq data indicates the existence of a previously unannotated exon (Figure 4B). Even with the mapping limitations imposed by the repeated structure of the gene, the Sashimi plots indicate that in each species, every exon encoding an EGF-like domain can probably be bypassed by an exon skipping event. Accordingly, the potential number of isoforms may be large, although limitations of the datasets, including varying read lengths, mean that additional testing is required to increase statistical confidence in this hypothesis. The level of heterogeneity suggested is, however, supported by the broad band detected on the northern blot analysis of mouse macrophages when mouse *Adgre1* cDNA was originally cloned, with the apparent size ranging over at least 1 kb (5).

### Analysis of the promoter and enhancer of *Adgre1*

**Figure 5:**
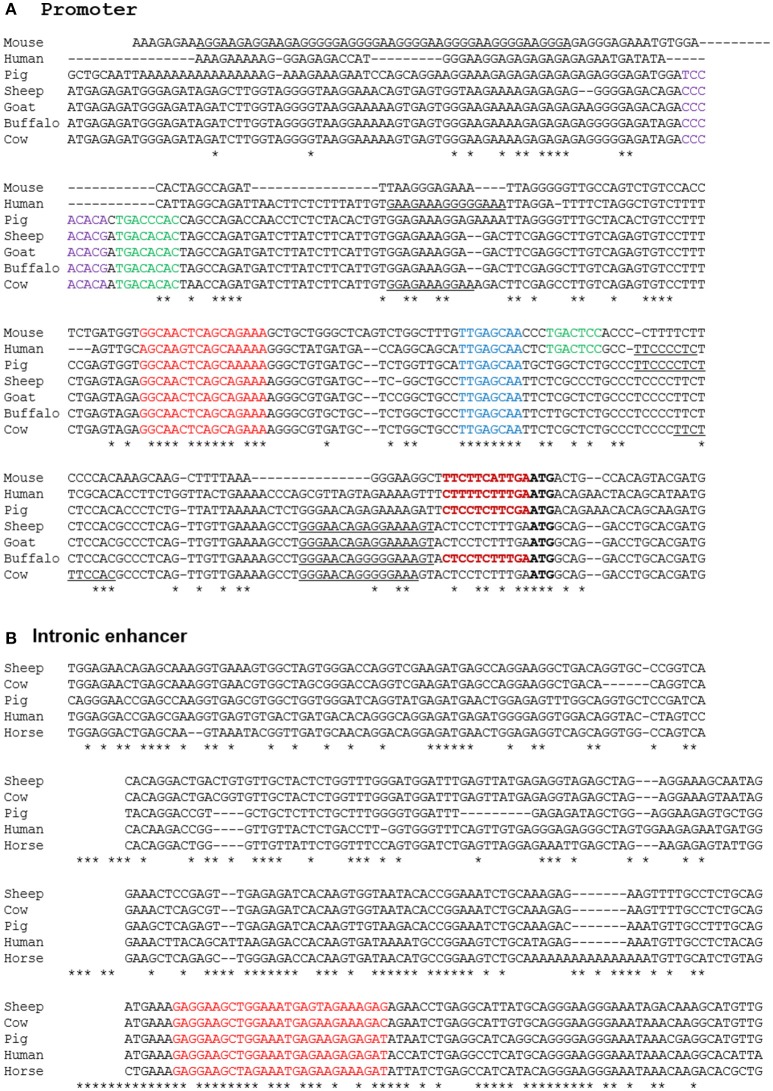
Alignment of promoter **(A)** and enhancer **(B)** regions of mammalian *ADGRE1* genes. Promoter sequences of the species shown were extracted from Ensembl initially based upon BLAST queries with either human or mouse sequences as the query, and then aligned using CLUSTALW. The ATG start codon in each species is in bold. The experimentally validated transcription start sites in mouse, human, pig and buffalo fall with the region immediately 5′ of the ATG (bold red). Conserved candidate regulatory elements highlighted include Maf (red), Runx1 (purple), AP1 (green), CEBP (blue). Purine-rich binding sites for PU.1 and/or other Ets factors are underlined. The intronic enhancer in the human gene lies at Chr19:6,892,130-8,892,530. This sequence was used to extract conserved sequences within the ADGRE1 locus of each species from Ensembl using BLAST. No hit was obtained on rodent genomes. The conserved PU.1/IRF8 motif is highlighted in red.

A number of alternative isoforms originally described for human and mouse ADGRE1 appear to encode N-terminally truncated proteins (63). However, a single major TSS region for *ADGRE1* is indicated from the FANTOM5 CAGE data from mouse and human macrophages, and we have confirmed the TSS region in CAGE data generated from pig BMDM (64) and from water buffalo (LL, RY, DAH, unpublished observations). Based upon the conserved expression in macrophages, we anticipated that regulatory elements would also be conserved. Accordingly, we extracted genomic sequences from Ensembl to compare the predicted transcription start sites and promoters for all of the species examined. The promoter in all species is TATA-less, and purine-rich, conforming to the pattern seen in many myeloid promoters in which the macrophage-specific transcription factor PU.1 is a key determinant of transcription initiation (65). The precise start site is conserved between the three species for which we have CAGE data (DAH, unpublished observations). Based upon these data, we can align the promoter sequences of mouse, rat, human, horse, pig, cattle, goat, sheep, and water buffalo as shown in Figure 5A. The promoter contains a perfectly-conserved binding site for C/EBP transcription factors, and for the Maf and MafB transcription factors which have both been shown, through the use of knockout mutations, to control F4/80 (*Adgre1*) expression in mice (66, 67). Analysis of the mouse *Adgre1* promoter revealed the presence of at least 9 purine-rich motifs containing the consensus GGAA core binding sites for PU.1 (68). As in humans, *ADGRE1* promoters from each of the large animals are purine-rich, but with a smaller number of the core GGAA motifs than observed in the mouse promoter, and with considerable variation in their location (Figure 5A). The FANTOM5 human CAGE datasets also reveal the presence of an enhancer within intron 2, consistent with a previously-published ChiP-seq analysis of human monocyte subsets (49). This element is conserved at the sequence level in large animal genomes (Figure 5B) and contains an identical motif to the PU.1/IRF8 responsive element of the human IFNß1 locus (69), which could contribute to the LPS-inducible expression of the gene. In overview, the conservation of regulatory elements is consistent with macrophage-specific expression in all of the species examined.

## Discussion

In this study, we have combined the generation of a new monoclonal antibody against pig ADGRE1 with detailed informatic analysis based upon RNA-Seq to gain further insight into the biology of ADGRE1 in mammals. The data in Figures 1–3 show clearly that *ADGRE1* is highly-expressed by pig monocytes, tissue macrophages and granulocytes and inducible during monocyte-macrophage differentiation from progenitors in the bone marrow. We demonstrated the utility of this new antibody for analysis of the response to agonists in pigs, such as the novel pig CSF1-Fc reagent (27, 44). ADGRE1 will provide a useful marker for dissection of infectious disease models in pigs, especially those of the lung where the pig is clearly more human-like (70).

The location of ADGRE1 expression in the pig revealed two major differences from the mouse. Mouse granulocytes express *Adgre1* mRNA, but do not translate the protein (20) whereas in pigs, ADGRE1 was highly-expressed on the surface of granulocytes in bone marrow. Consistent with the protein expression (45), *Adgre1* mRNA is very low in mouse alveolar macrophages (71) and we have found the same at the mRNA level in rats (CP, DAH, manuscript in preparation). By contrast, ADGRE1 protein was very highly-expressed in both alveolar and interstitial macrophages in the pig, and at least at the mRNA level, this pattern appears to be conserved in all the large animals including humans.

We summarized evidence from multiple datasets and platforms that the expression pattern we have dissected in pigs is shared with humans and other large animals. *ADGRE1* mRNA is not, as previously suggested, restricted in its expression to eosinophils or even enriched in those cells in humans. Indeed, the gene is a marker for differentiation of the CD16^++^ monocyte subset in human peripheral blood and is also highly-expressed in many tissue macrophage populations, notably those of the lung. Expression of *ADGRE1* in macrophages in all large animals is consistent with the conservation of promoter and enhancer elements across species. Nevertheless, where *Adgre1* mRNA and protein were induced by CSF1 in rodent macrophages, in the large animals *ADGRE1* mRNA expression in macrophages was down-regulated, compared to monocytes and, in some species, apparently inducible by LPS (Table S1). The pattern of regulation of *ADGRE1* mRNA, as well as its genomic location, suggests a possible role in genetic susceptibility to inflammatory bowel disease (30). Thus, although some promoter elements that drive macrophage expression are conserved (Figure 5A), there is also evidence for evolution of transcriptional regulatory mechanisms within selected macrophage populations.

The two previous reports of specific *ADGRE1* mRNA and protein expression in eosinophils are difficult to reconcile with our analysis. Both reports used the same set of PCR primers, amplifying a short product from bases 1,408 to 1,490 in the cDNA sequence. To explain the apparent selective binding of anti-ADGRE1 antibodies to eosinophils, there is the formal possibility that the *ADGRE1* protein is selectively translated in these cells. Alternatively, the antibodies may cross-react with related calcium-binding EGF domain-containing molecules such as ADGRE3 which are actually strongly enriched in granulocytes, or with a specific glycoform of ADGRE1 produced by eosinophils. Whatever the explanation, it would clearly be worthwhile to make and test additional anti-human ADGRE1 reagents.

Aside from differences in gene expression profiles, ADGRE1 also varies greatly at the protein sequence level between species, including differences in the numbers of the EGF-like calcium binding domains. Within a species, the EGF-like domains also differ in sequence from each other. The mouse *Adgre1* cDNA described originally encoded 7 EGF-like calcium binding domains. Subsequent analysis of both mouse cDNAs identified at least 5 variants differing in the inclusion of individual domains. These included variants that lack exon 4, which contains the likely site for glycosaminoglycan attachment (5). Human *ADGRE2* shows evidence of both alternative splicing of the EGF-like domains and regulated glycosylation (60). There is also some evidence of alternative splicing of the human *ADGRE1* transcript. Sequencing of multiple full-length mouse macrophage cDNAs by the FANTOM consortium (72) identified further variants, apparently containing any combination of the EGF-like domains and including a possible secreted isoform. The longest cloned human *ADGRE1* cDNA in Ensembl encodes 6 EGF-like calcium binding domains, but our analysis indicates that there are actually 7 domains, as in rodents and pigs. By contrast to these species, the longest predicted cDNA encoding ADGRE1 in other species are considerably larger. The longest Ensembl sheep cDNA encodes 1,259 amino acids and 13 calcium binding EGF-like domains but the RNA-Seq data suggests there is at least one additional exon. The predicted protein for the goat is only 905 amino acids, which is clearly incorrect since the longest Ensembl-predicted sheep cDNA maps in its entirety to the goat genome (not shown). The longest Ensembl-predicted cattle protein is 1,148 amino acids and contains 12 predicted EGF-like calcium binding domains. However, the RNA-Seq data indicates the existence of at least two additional exons and the entire sheep cDNA maps to the cattle genome. Longer forms of ADGRE1 are also predicted by Ensembl for cat (1,101 amino acids) and dog (1,162 amino acids), but here again, the entire sheep cDNA maps contiguously to the dog and cat genome (not shown). In overview, the longer forms in ruminants, horses, cats, and dogs probably encode 14 EGF-like domains arising from a duplication of the 7-domain structure of rodent, pig and human. Five of the additional domains contain the acidic motif (most commonly CEDxDEC) implicated in calcium binding which is present in 5 of the 7 EGF-like domains of mouse *Adgre1* (5). Splicing analysis of the RNA-Seq data derived from BMDM (Figures 4A,B) from each of these species reveals that as in mouse, human and pig, each of the EGF-like domains, encoded by single exons, in frame, exhibit evidence of exon-skipping, with the potential to produce numerous different isoforms. The protein sequence of ADGRE1 is highly divergent amongst species. For example, mice and pigs have only 58% identity, and a dN/dS ratio of only 0.24 (obtained via Ensembl using methods detailed at https://www.ensembl.org/info/genome/compara/homology_method.html). Almost all coding differences reside in the extracellular domain. In the EGF-like domains, only the structural and calcium-binding motifs are conserved. This may imply that there is no evolutionary/functional constraint on the sequence of these domains other than preservation of structure. The human exome database (http://exac.broadinstitute.org) reveals that *ADGRE1* (*EMR1*) is also polymorphic in human populations, with numerous non-synonymous protein-coding variants with high allele frequencies. Interestingly, a missense splice donor site, with a population allele frequency of 0.38, might explain the apparent lack of one exon in the longest current human cDNA. Alternatively, the rapid evolution of protein-coding sequences, and gene duplication, has long been recognized as a feature of immune-associated genes (73). Both within species polymorphism and accelerated between species divergence of innate immune genes was highlighted in detailed analysis of the pig immunome (74).

Hamann et al. (7) reviewed the evidence that members of the adhesion G protein coupled receptor family [*ADGRE1, ADGRE2, ADGRE3, ADGRE4*, and CD97 (aka *ADGRE5*)] are modulators of immune cell function. Analysis of the function of this gene family has been constrained by the lack of knowledge of likely ligands. The only evidence comes from ADGRE2 which was reported to bind selectively to the proteoglycan chondroitin sulfate (75). Interaction with various glycosaminoglycans (GAG) at portals of entry is a very common feature of microbial pathogenesis (76). Conceivably, host GAG might act as opsonins to bind pathogens to innate immune cells through the ADGRE family. In the case of ADGRE1, no ligand has been identified, but the pig ADGRE1 fusion protein we used as an immunogen in the current study will facilitate future binding studies. The diversification of the extracellular domain through evolutionary selection and splicing, and high expression in lung macrophages in pigs, humans and other large animals, support the hypothesis that ADGRE1 has evolved in response to selection by pathogens, and participates in the host defense. A recent report demonstrated that cross-linking with anti-ADGRE2 antibody produced a pro-inflammatory signal in a human monocytic cell line (77). To our knowledge, the widely-used anti-mouse ADGRE1 antibody, F4/80, has not been shown to generate a detectable signal when added to macrophages. The availability of anti-ADGRE1 antibodies for the pig will enable further functional studies in this species that may translate to humans and other species.

## Author contributions

LW, LL, and SB contributed equally to the study and carried out experimental work, data and sequence analysis. AR, RY, ZL, MM, CM, KS, EC, KI, and CP contributed to experimental work and data analysis. DH conceived the idea and secured funding for the study. JH and DH supervised the experimental work and contributed to data analysis. All authors contributed to the manuscript.

### Conflict of interest statement

The authors declare that the research was conducted in the absence of any commercial or financial relationships that could be construed as a potential conflict of interest.

## Abbreviations

ADGRE1: Adhesion G protein coupled receptor E1
BMDM: bone marrow-derived macrophages
CSF1: colony-stimulating factor 1
EGF: epidermal growth factor
HI-FCS: heat inactivated fetal calf serum
MDM: monocyte-derived macrophages
PBS-T: PBS containing 0.05% Tween-20.
